# Postbiotic heat-killed lactobacilli modulates on body weight associated with gut microbiota in a pig model

**DOI:** 10.1186/s13568-022-01424-8

**Published:** 2022-06-29

**Authors:** Sangdon Ryu, Hyunjin Kyoung, Kyeong Il Park, Sangnam Oh, Minho Song, Younghoon Kim

**Affiliations:** 1 Department of Agricultural Biotechnology and Research Institute of Agriculture and Life Science, Seoul National University, 08826 Seoul, Korea; 2 Division of Animal and Dairy Science, Chungnam National University, 34134 Daejeon, Korea; 3 Department of Functional Food and Biotechnology, Jeonju University, 55069 Jeonju, Korea

**Keywords:** Heat-killed *Ligilactobacillus salivarius*, Postbiotics, gut microbiota, Anti-obesity, Lipid metabolism

## Abstract

For decades, *Lactobacillus* has been extensively used as beneficial probiotics because it positively effects on the intestinal health of the host and has been studying its possible serve to treat obesity as well as various diseases. This research aimed to investigate the effects of heat-killed *Ligilactobacillus salivarius* strain 189 (HK LS 189) supplementation on anti-obesity and gut microbiota. A total of 48 pigs were fed either a basal diet or a diet supplemented with HK LS 189 for 4 weeks. The impact of HK LS 189 supplementation on the composition and function of the intestinal microbiota was revealed by 16 S rRNA gene sequencing. HK LS 189 supplementation significantly decreased growth performance. Moreover, HK LS 189 supplementation altered the gut microbiota of the pigs by decreasing the proportion of *Prevotella* and increasing the proportion of *Parabacteroides.* Beta-diversity analysis showed a significant difference between the two groups. The results support the potential use of HK LS 189 for its anti-obesity effect in pigs through modulation of the gut microbiota. Furthermore, we found changes in the functional pathways of the gut microbiota. The functional pathway study indicated that metabolism and lipid metabolism differed between the two groups. Our data may contribute to understanding the potential use of postbiotic supplementation with HK LS 189 for improving the anti-obesity effects.

## Introduction

Obesity is a chronic metabolic disease including cardiovascular diseases, type 2 diabetes, and liver diseases and a major health concern growing epidemic worldwide (Piché et al. 2020). Therefore, various studies have been conducted with a lot of interest related to the anti-obesity effect. The gut microbiota is a dynamic ecosystem with diverse symbiotic bacterial populations that influence immunity, and overall health. Interactions with the gut microbiota influence the replacement rate of intestinal epithelium cell population and thus effect nutrient absorption and gut microbiota may contribute to anti-obesity (Duranti et al. 2017; Rosenbaum et al. 2015).

Probiotics have attracted increasing attention because they are safe, their roles in modulating the gut microbiota, and demonstrate significant beneficial effects (Ouwehand et al. 2002). Generally, probiotics change the composition, function, and metabolites of gut microbiome and responds to the body by the immune signal transduction (Galdeano et al. 2019). However, on the other hand, because of probiotics directly impact the gut microbiota and have instability, recently, there has been growing interest in postbiotics (Żółkiewicz et al. 2020). The concept of postbiotics is nonviable microbial cells that benefit human or animal consumers and based on the observation that the beneficial effects of the microbiota are mediated by the secretion of various metabolite. Previous studies have shown that heat-killed *Lactobacillus* spp. have an anti-adiposity effect, ameliorate obesity in HFD-induced obesity model and can alleviate obesity by regulating gut microbiota-mediated AMPK and NF-κB activation and SIRT-1 expression (Jang et al. 2019; Uchinaka et al. 2018). Therefore, not only live cell but also heat-killed cell could improve obesity or obesity-associated disorders.

In this study, we hypothesized that the targeted strains of postbiotic heat-killed lactobacilli have diverse influences on the gut microbiota proportion and growth performance in pig model. Pigs are very similar to humans in gastrointestinal (GI) function and composition, making them an ideal non-primate large animal model (Roura et al. 2016). Pig and human GI microbiota have a bacterial composition that is 96% similar to each other in functional pathways (Lim et al. 2019). Although there are differences among butyrate producers, studies have reported that the composition of the fecal microbiome of pigs is similar to that of humans (Kobayashi et al. 2020; Xiao et al. 2016). In fact, studies have been reported on how various diets, including high-fat diet, affect the gut microbiota in a pig model (Heinritz et al. 2016a, b). Therefore, the objective of this study was to explore the effects of postbiotic heat-killed lactobacilli on the anti-obesity effects and intestinal microorganisms in pig model. Given this, the information we found could provide valuable evidence based on gut microbiota analysis for explaining the anti-obesity influence of postbiotic heat-killed lactobacilli.

## Materials and methods

### Ethics statement

The experimental protocol was reviewed and approved by the Animal Care and Use Committee of Chungnam National University, Daejeon, Korea (Approval Protocol # 202,006 A-CNU-090).

### Preparation of probiotics

*Ligilactobacillus salivarius* strain 189 (isolated from Korean healthy infant feces and deposited to the Korean Agriculture Culture Collection Center [Deposition number: KACC 22,719]) was cultured in De Man-Rogosa-Sharpe broth (MRS; BD Difco, Sparks, MD, USA) for 24 h at 37 °C. The bacterial pellets were collected, washed twice and resuspended with saline. And then, heat-killed *L. salivarius* (HK LS 189) were prepared by treatment at 90 °C for 15 min, and the absence of colony formation was determined by incubation for 48 h at 37 °C. The concentration of HK LS 189 in the dry product was ca. 1.0 × 10^10^ cells/well.

### Animal administrations and sampling

In a randomized completely block design [block = body weight (BW)], a total of 48 pigs [Duroc x (Landrace x Yorkshire); average initial BW = 76.62 ± 2.59 kg] were assigned to 2 dietary treatments (6 replicates/treatment; 4 barrows/pen). Dietary treatments were (1) a basal diet based on a corn-soybean meal (CON) and (2) a basal diet supplemented with 0.2% HK LS 189. The experimental period was for 4 weeks. The basal diet was formulated to meet or exceed the nutrient requirements according to the NRC (2012) for finishing pigs (Table 1). All pigs were allowed *ad libitum* access to diet and water and were housed in the same sized pen (5.95 m × 10.5 m) with environmentally controlled conditions (ambient temperature of 15–20 °C; 12-h light/dark cycle) by an automatic mechanical system throughout the experimental period.

**Table 1:** Composition of the basal diet for finishing pigs (as-fed basis)

Items^1^	Basal diet
Ingredient (%)	
Corn,	70.80
Soybean Meal, 44%	25.00
Soybean Oil	2.00
Limestone	1.00
Iodized Salt	0.20
Vit–Min Premix^1)^	0.40
Total	100
Calculated energy and nutrients	
ME, kcal/kg	3,399
CP, %	16.81
Ca, %	0.59
P, %	0.47
Lys, %	0.87
Met, %	0.28
TSAA, %	0.58
Thr, %	0.64
Trp, %	0.19

^1^Provided per kilogram of diet: vitamin A, 12,000 IU; vitamin D3, 2,500 IU; vitamin E, 30 IU; vitamin K3, 3 mg; D-pantothenic acid, 15 mg; nicotinic acid, 40 mg; choline, 400 mg; vitamin B12, 12 µg, Fe, 90 mg from iron sulfate; Cu, 8.8 mg from copper sulfate; Zn, 100 mg from zinc oxide; Mn, 54 mg from manganese oxide; I, 0.35 mg from potassium iodide; and Se, 0.30 mg from sodium selenite

The individual BW of pigs and residual feed after supply were weighed and recorded on the initial and final days of the study to evaluate the average daily gain (ADG), average daily feed intake (ADFI), and feed efficiency (gain:feed; gain to feed ratio) for their growth performance. Fecal samples were collected from all pigs in each dietary treatment on the last day of the study by rectal palpation using a sterile cotton swab to evaluate their gut microbiota. The fecal samples were placed in sterile tubes and stored at -80 °C until further metagenome analysis.

### Microbiota analysis

DNA was extracted from fecal samples using the Powerfood Microbial DNA Isolation kit (Mo Bio Laboratories, Inc., Carlsbad, CA, USA) according to the manufacturer’s instructions. Each DNA sample was adjusted to a concentration of 1 ng/µL and subjected to PCR according to the 16S Metagenomic Sequencing Library protocols (Illumina, San Diego, CA, USA). The V4 region of the 16S rRNA genes (primer set: forward, 5′ -TCG TCG GCA GCG TCA GAT GTG TAT AAG AGA CAG GTG CCA GCM GCC GCG GTA A-3’; reverse, 5′ -GTC TCG TGG GCT CGG AGA TGT GTA TAA GAG ACA GGG ACT ACH VGG GTW TCT AAT-3′) was analyzed using the Illumina MiSeq platform (Illumina, San Diego, CA, USA). After the concentrations of the index PCRs were measured by PicoGreen (Invitrogen, Carlsbad, CA, USA), equimolar PCR amplicons were pooled and sequenced using the MiSeq system platform (Macrogen, Seoul, South Korea) based on the standard Illumina sequencing protocols.

Fastq files obtained from MiSeq paired-end sequencing data were analyzed using Mothur (v. 1.45.3) (Schloss et al. 2009). Briefly, error removal was performed using a nonaligned screening sequence with the Silva database (version 138) (Pruesse et al. 2007) to merge rare sequences into large sequences. Chimeric sequences were detected using Vsearch (Rognes et al. 2016). Taxonomic classification was analyzed using the Greengenes-formatted database (DeSantis et al. 2006) released in 2013 to eliminate sequences not categorized as archaea or mitochondria. Singletons were removed using the Mothur subroutine “split.abund” (Unno 2015). The operational taxonomic units (OTUs) were classified using the distance 0.03 calculation (97% sequence similarity) and binned using the opti clust algorithm. UniFrac distance was analyzed to assess the differences among sites based on phylogenetic information using PERMANOVA tests (Lozupone et al. 2011). We visualized differences in the microbial community composition using unweighted and weighted UniFrac distances through nonmetric multidimensional scaling analysis (NMDS). Each symbol on an NMDS plot shows the total microbial community of each sample. Symbols closer together have more similar microbiota compositions, while those farther apart have less similarity.

### Bioinformatics analyses

16 S rRNA sequencing was performed for all samples, and the alpha-diversity and relative abundance of OTU analyses for phylum and genus were performed using the MicrobiomeAnalyst web-based platform (Chong et al. 2020). To identify differential taxa, a linear discriminant analysis effect size (LEfSe) (Segata et al. 2011) analysis was performed using an online tool (https://huttenhower.sph.harvard.edu/galaxy/). To predict functional pathways in the microbiome, PICRUSt and Kyoto Encyclopedia of Genes and Genomes (KEGG) (level 2) were used to generate a list of functional genes predicted to be present in the sample. PICRUSt (Langille et al. 2013) was performed using an online tool (https://huttenhower.sph.harvard.edu/galaxy/). The relative abundance difference of bacteria and significantly different KEGG pathways between groups were identified using STAMP v0.2.1.3 (Parks et al. 2014) by extended error bar plot. The datasets generated and/or analyzed during this study are available in the BioProject repository, http://www.ncbi.nlm.nih.gov/bioproject/830296.

### Statistical analysis

All data for growth performance were analyzed with the PROC GLM procedure of SAS (SAS, Carry, NC, USA) in a completely randomized design with the pen as an experimental unit. The model for growth performance included dietary treatment as a fixed effect. Statistical differences and tendencies were considered at *p* < 0.05 and *p* < 0.10, respectively. R statistical software (version 4.0.5) was used for the microbial diversity analysis. Comparison of bacterial alpha-diversities (the Shannon, Chao1, and Simpson indices) was performed by nonparametric one-way analysis of variance (Kruskal–Wallis test) followed by subsequent Tukey’s posthoc analysis if there was a significant difference (p < 0.05). For UniFrac, we used the phyloseq package (McMurdie and Holmes 2013) to calculate the distances and then the vegan package (Oksanen et al. 2013) to run permutational multivariate analysis of variance (PERMANOVA) tests to partition the variation between groups (Lozupone et al. 2011). Also, environmental fitting (envfit) function from the vegan package was used to show the correlation between the environmental variables and the bacterial communities onto the ordination space.

## Results

### Effects of feeding HK LS 189 on pig growth performance

The growth performance of the pigs after 4 weeks of feeding with HK LS 189 supplementation is summarized in Table 2. We found significant differences in the final body weight (*p* < 0.05), ADG (*p* < 0.05), ADFI (*p* < 0.10), and gain:feed (*p* < 0.10) between pigs in the control and HK LS 189 groups (*p* < 0.05), indicating that HK LS 189 supplementation affects growth performance. These results indicate that HK LS 189 induced a significant decrease in growth performance.

**Table 2:** Effect of heat-killed *L. salivarius* on the growth performance of finishing pigs

	Dietary treatments^2^			
Item	Control	Heat-killed ***L. salivarius***	SEM	***p*** value^1^
Initial Body Weight (kg)	76.67	76.58	2.49	0.847
Final Body Weight (kg)	106.35	95.34	2.98	0.015
Average Daily Gain (kg/d)	1.06	0.67	0.12	0.036
Average Daily Feed Intake (kg/d)	2.34	2.81	0.18	0.075
Gain:Feed (kg/kg)	0.453	0.238	0.075	0.097

^1^Values are expressed as the mean of at least 6 replicates (4 pigs/pen) and as the mean ± standard error of the mean (SEM)

^2^Control = basal diet, *L. salivarius* = basal diet + 0.2% heat-killed *L. salivarius*

### Alpha- and beta-diversity in the fecal microbiota of pigs

**Fig. 1:**
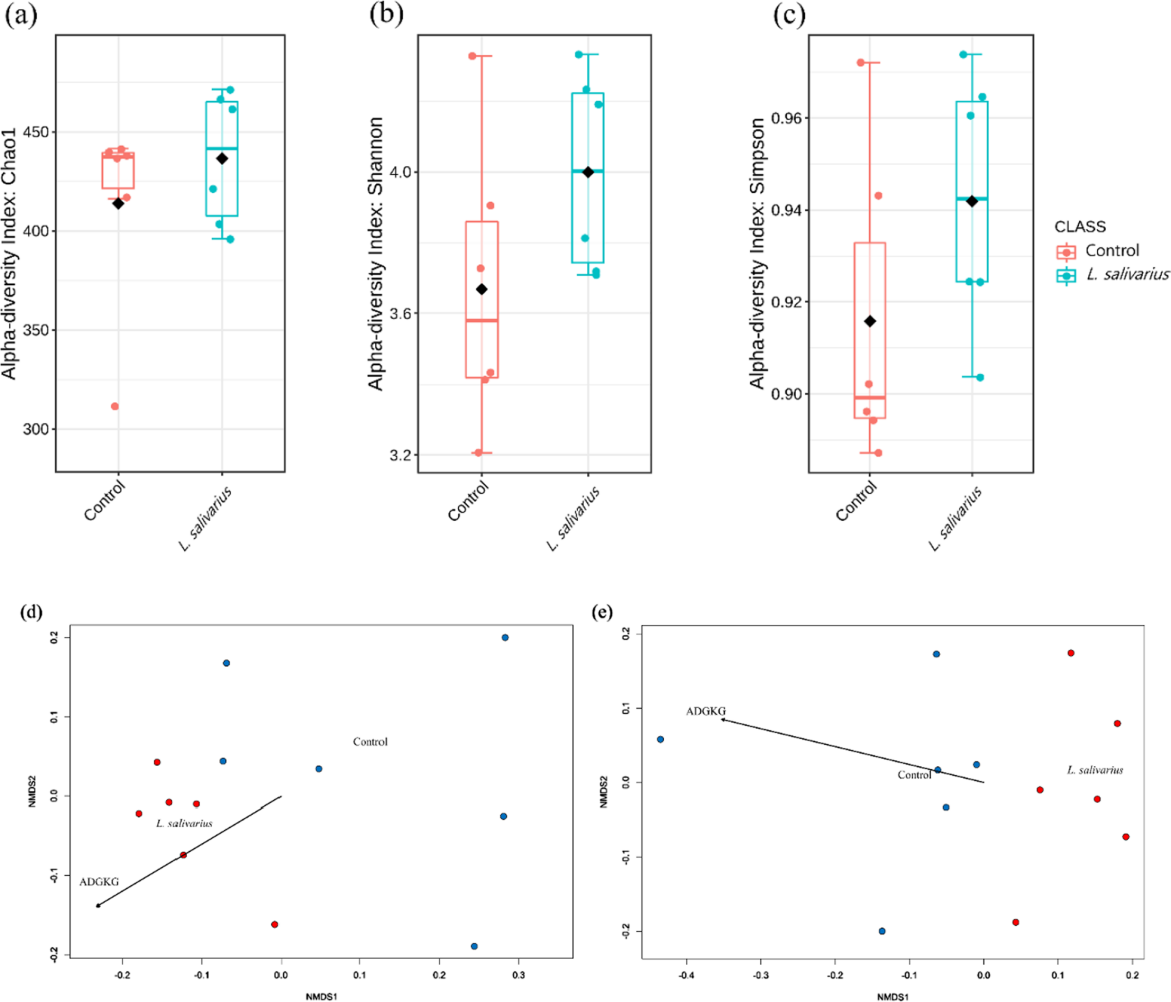
Microbial richness and diversity. Comparison of the alpha-diversity indices **a** Chao1, **b** Shannon, and **c** Simpson and the beta-diversity indices of each group in weighted **d** and unweighted **e** NMDS plots of UniFrac distances of samples in two groups (control and heat-killed *L. salivarius*)

Alpha-diversity of the microbiota was observed using the Chao1, Shannon, and Simpson indices. The bacterial richness is represented by the Chao1 index. The Chao1 index did not differ significantly between the control and HK LS 189 groups. The Shannon and Simpson indices are diversity indices that provide important information on richness and evenness within the community. As with the Chao1 result, the Shannon and Simpson indices did not differ significantly. Overall, there was no significant difference between the two groups in alpha-diversity (Fig. 1a**–**c).

Next, we examined beta-diversity distances between samples using weighted and unweighted UniFrac distances. Weighted and unweighted UniFrac distances were visualized by NMDS analysis and were analyzed by PEMANOVA. A significant difference in weighted UniFrac was observed between the control and HK LS 189 groups (p = 0.01). Similarly, significant differences in unweighted UniFrac distance were found between the control and HK LS 189 groups (p = 0.003) (Fig. 1d, e). With beta-diversity analysis, we discovered that HK LS 189 group samples clustered separately from samples of the control group.

### Gut microbiota composition

**Fig. 2:**
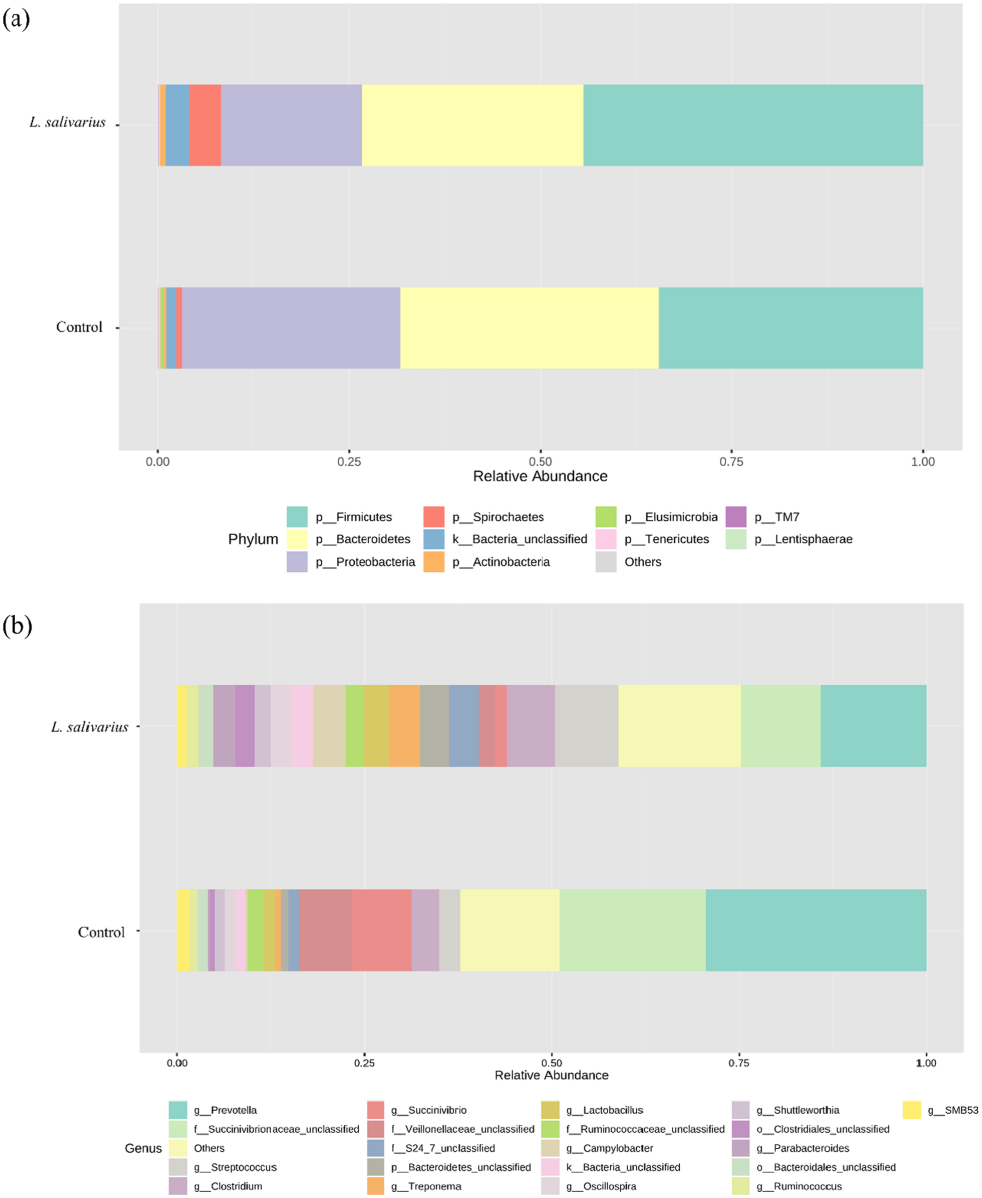
The effect of heat-killed *L. salivarius* supplementation on the fecal microbiota composition at the phylum and genus levels. **a** The color corresponds to phylum, and **b** the color corresponds to genus

All 9,876,756 high-quality sequencing reads from 12 samples were clustered into OTUs at the 97% similarity level. A total of 1,693 OTUs were identified, which were then classified into taxonomic groups at a threshold of 80%. The sequencing depth was suggestive of the sufficient sequencing depth for metagenomic analyses according to the Good’s coverage scores for both the control and HK LS 189 groups (99.7% and 99.8%, respectively). At the phylum level, the predominant bacterial taxa were *Firmicutes*, *Proteobacteria*, and *Bacteroidetes*, followed by *Spirochaetes* and *Actinobacteria* in both groups (Fig. 2a). The relative abundance of the predominant phylum was not significant in either the control or HK LS 189 groups. Still, the relative abundance of *Lentisphaerae* (0.07%) in the HK LS 189 groups was significantly higher (P = 0.021) than that in the control group (0.02%). The abundance of *Cyanobacteria* (0.09%) was significantly higher (P = 0.025) in the control group than in the HK LS 189 group (0.01%).

**Fig. 3:**
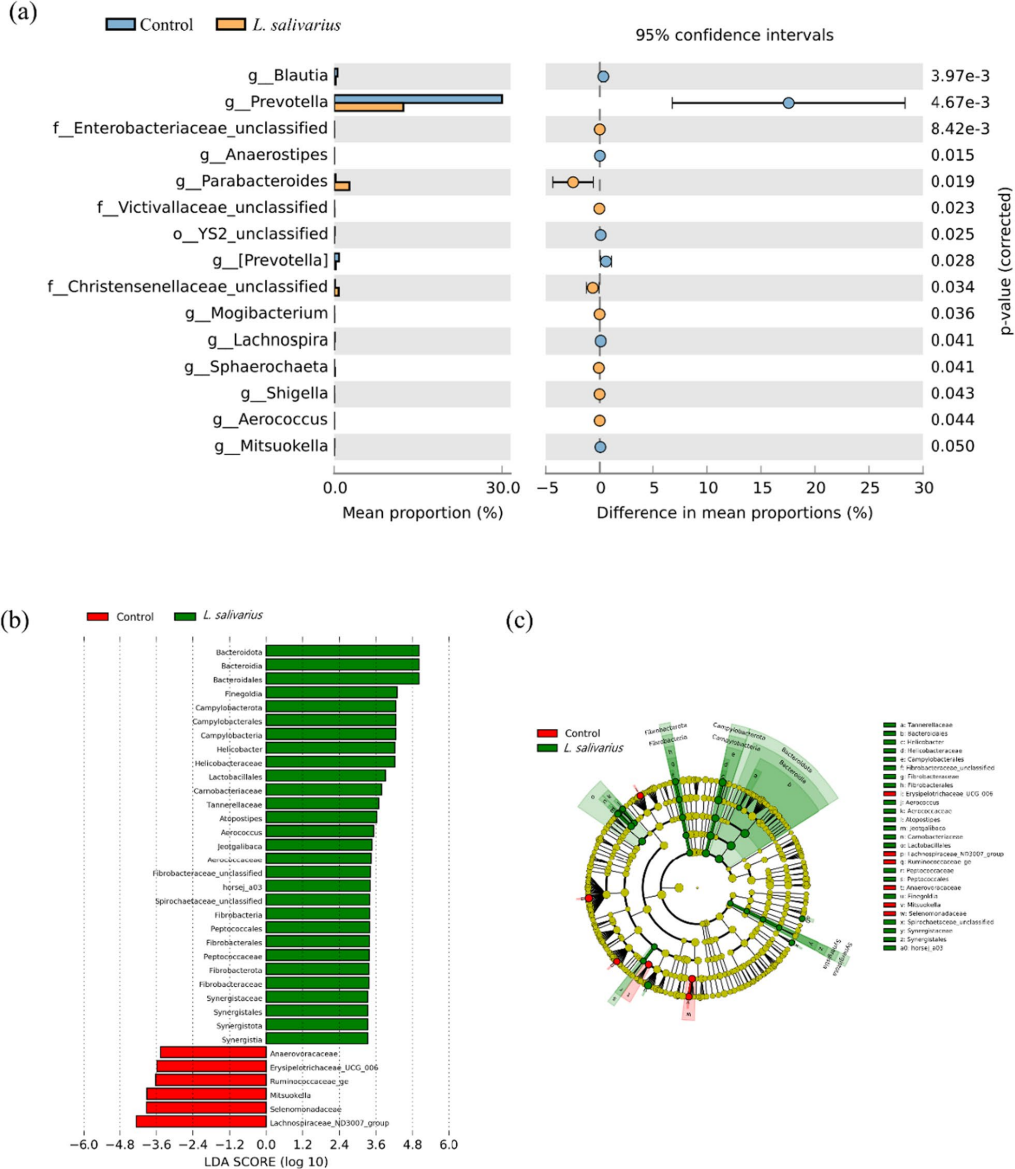
The relative abundance of bacterial community composition. Comparison of the gut microbiota between the control group and the *L. salivarius* group at the genus level with significantly different abundances (**a**). Taxonomic feature identification between the control and heat-killed *L. salivarius* groups using linear discriminant analysis effect size (LEfSe) analyses: **a** histogram and **b** cladogram. Significance was present at p < 0.05 for the LEfSe analyses for both the Kruskal–Wallis and Wilcoxon tests, and the minimum linear discriminant analysis (LDA) score was set to 2.0

At the genus level, the predominant bacterial taxa were *Prevotella*, *Succinivibrionaceae*_unclassified and *Veillonellaceae*_unclassified, followed by *Clostridium* and *Streptococcus* in both groups. *Prevotella* was the most abundant genus in both groups. Supplementation with HK LS 189 led to a significant decrease in *Prevotella* (30.02% vs. 12.46%; *p* = 0.005), *Blautia* (0.59% vs. 0.26%; *p* = 0.004), *Lachnospira* (0.09% vs. 0.01%; *p* = 0.04), YS2_unclassified (0.09% vs. 0.01%; *p* = 0.025), *Mitsuokella* (0.05% vs. 0.003%; *p* = 0.049), and *Anaerostipes* (0.005% vs. 0.0002%; *p* = 0.015) (Figs. 2b and 3a).

Next, we identified taxonomic biomarkers of the control and HK LS 189 groups in the gut microbiota of the pigs using LEfSe analysis (Fig. 3b, c). A total of 35 differentially enriched bacterial colonizers (29 in HK LS 189 and 6 in control) were identified. The enriched abundances of *Bacteroidota, Bacteroidia, Bacteroidales, Finegoldia, Campylobacterota, Campylobacterales, Campylobacteria, Helicobacter, Helicobacteraceae, Lactobacillales, Carnobacteriaceae, Tannerellaceae*, and *Atopostipes. Aerococcus, Jeotgalibaca, Aerococcaceae, Fibrobacteraceae_unclassified, horsej_a03, Spirochaetaceae_unclassified, Fibrobacteria, Peptococcales, Fibrobacterales, Peptococcaceae, Fibrobacterota, Fibrobacteraceae, Synergistaceae, Synergistales, Synergistota*, and *Synergistia* were identified as taxonomic markers for the HK LS 189 group (p < 0.05). In contrast, *Lachnospiraceae_ND3007_group, Selenomonadaceae, Mitsuokella, Ruminococcaceae_ge, Erysipelotrichaceae_UCG_006*, and *Anaerovoracaceae* were identified as taxonomic features of the control group (p < 0.05). Using the LEfSe method, we found that HK LS 189 supplementation causes a regulatory effect on the gut microbiota of pigs.

### Differential KEGG functional pathways in the gut microbiota

**Fig. 4:**
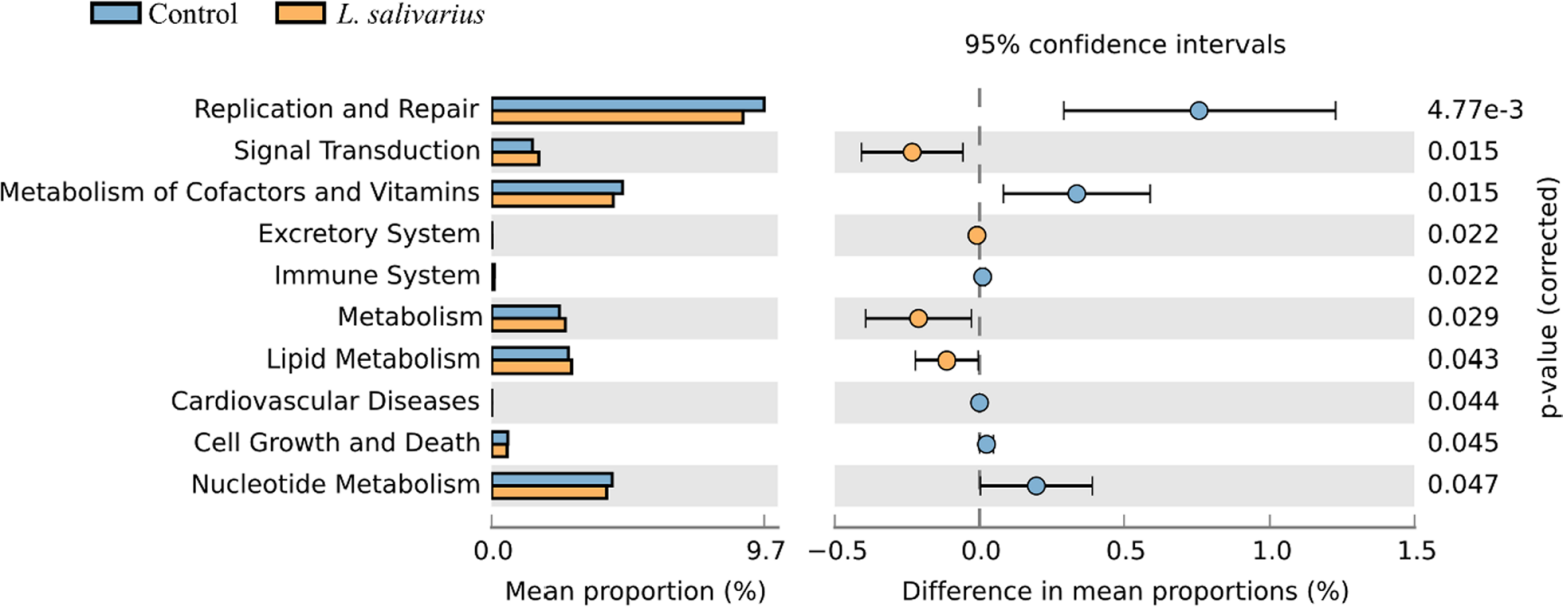
KEGG pathways predicted in the gut microbiota of the control and heat-killed *L. salivarius* groups using PICRUSt.Statistical analysis was carried out using STAMP software.

To determine the differences in microbial pathways between the HK LS 189and control groups, we evaluated the microbial community functional profiles using PICRUSt analysis and data visualization using STAMP (Fig. 4). Among the 41 affiliated KEGG pathways (at level 2), 10 were shown to achieve a statistically significant difference in the HK LS 189 group (p < 0.05), and 66 KEGG pathways of 328 affiliated KEGG pathways in level 3 were significantly enriched in the HK LS 189group (p < 0.05). Notably, pathways related to signal transduction (1.46% vs. 1.69%, P < 0.05), the excretory system (0.01% vs. 0.02%, P < 0.05), metabolism (2.39% vs. 2.6%, P < 0.05), and lipid metabolism (2.71% vs. 2.83%, P < 0.05) were significantly elevated in the HK LS 189 group.

## Discussion

In this study, we investigated whether HK LS 189 supplementation affects pigs’ growth performance and microbiota. In our experiment, when pigs were fed HK LS 189, the growth performance was reduced compared to the control group. Next, the microbiota analysis did not show a significant difference in alpha-diversity, but the beta-diversity analysis did show a significant difference. As a result of microbiota composition analysis, three phyla (Firmicutes, Bacteroidetes, and Proteobacteria) at the phylum level showed abundances of 97.7% and 87.2% in the control group and HK LS 189 group, respectively. Additionally, only *Lentisphaerae* and *Cyanobacteria*, which account for rare abundances, showed significant differences between the two groups. At the genus level, there were significant differences in 15 genera; in particular, the relative abundance of *Prevotella* showed the most significant difference between the two groups. In *Prevotella*, it was found that the control group accounted for 17.56% more than the HK LS 189 group.

Supplementation with *Lactobacillus* species promotes mucus secretion in the small intestine and helps the growth of intestinal microorganisms. In fact, *L. salivarius* is a potential probiotic reported to have the ability to enhance the immune system, attenuate intestinal inflammation, and exert antibacterial activity against pathogenic bacteria, among other effects to date (Alard et al. 2016; Lee et al. 2020; Pothuraju and Sharma 2018). Despite the positive effects of probiotics, including *L. salivarius*, nonviable microbial cells may have safety benefits over probiotics because they decrease the risk to the host of an imbalanced or compromised immune system. For this reason, recent studies on postbiotics have been actively conducted. Studies have been reported on the differences in the effects of live cells and heat-killed cells on immune function and the intestinal environment, respectively (Jang et al. 2018; Sugahara et al. 2017). Our experimental results provided a different aspect from the results of a previous study. Contrary to the results of earlier studies in pigs, HK LS 189 supplementation decreased the growth performance in this study. As such, the *L. salivarius* strain used in our study may also have a function similar to that of various probiotics with anti-obesity effects. Although it was not a study on pigs, a recently published paper showed that supplementation of *L. salivarius* to mice had an anti-obesity effect. Liang et al. (Liang et al. 2021) explained that *L. salivarius* exhibits an anti-obesity effect by inhibiting food intake and causing changes in gut microbiota by promoting peptide YY secretion. In this study, although we did not elucidate the specific mechanism of the anti-obesity effect exhibited by HK LS 189 supplementation, gut microbiota analysis revealed that HK LS 189 supplementation had an apparent effect on the regulation of gut microbiota in pigs.

A high proportion of *Prevotella* in the gut microbiota is associated with various metabolic diseases, such as obesity, insulin resistance in nondiabetic people, hypertension, and nonalcoholic fatty liver disease. In particular, some studies have found a positive correlation between the proportion of *Prevotella* and obesity (León-Mimila et al. 2018; Moreno-Indias et al. 2016). In addition, a recent study reported that *Prevotella copri* promotes fat accumulation in pigs fed formula diets (Chen et al. 2021). Similar to previous studies, we confirmed that the abundance of *Prevotella* was significantly reduced in the HK LS 189 group compared to the control group. At the same time, the growth performance was reduced compared to the control group. Among the genera, *Parabacteroides* was significantly increased in the HK LS 189 group compared to the control group. *Parabacteroides*, such as *Prevotella*, have also been associated with obesity in some studies. In particular, *Parabacteroides goldsteinii* and *Parabacteroides distasonis* showed anti-obesity effects when they were predominant in the intestine (Wu et al. 2019) and alleviated pathogenesis by an anti-inflammatory response through gut microbiota modulation (Lai et al. 2021). At the genus level, we found that HK LS 189 supplementation caused the significant enrichment of *Bacteroidota*, *Bacteroidia*, and *Bacteroidales* belonging to the Bacteroidetes phylum through LEfSe analysis. It is generally accepted that obesity is accompanied by a decrease in *Bacteroidetes* (Ley et al. 2006; Pérez-Matute et al. 2015). These findings suggest that HK LS 189 supplementation influenced gut microbiota. The proportion of *Prevotella* decreased, and the proportion of *Parabacteroides* and *Bacteroidetes* increased, resulting in reduced growth performance.

Finally, our PICRUSt analyses revealed that HK LS 189 supplementation also adjusted the metabolic functions of the gut microbiota. The HK LS 189 group was enriched in several KEGG pathways, including signal transduction, the excretory system, metabolism, and lipid metabolism. The alteration of microbial composition is always accompanied by significant functional modification. In a past study, the most common postbiotics were short-chain fatty acids (SCFAs), namely, acetate, propionate, and butyrate, as well as peptides, enzymes, teichoic acids, and vitamins. Among the factors mentioned above, SCFAs induce thermogenesis in brown adipose tissue and browning in white adipose tissue, so they can be used as candidates for preventing and treating obesity (Li et al. 2018). Our experimental results showed that HK LS 189 supplementation could increase the abundance of metabolism and lipid metabolism pathways, which are associated with anti-obesity effects (Li et al. 2020; Wu et al. 2020). Therefore, it is thought that the regulatory action on lipid metabolism of SCFAs derived from *L. salivarius* was shown to have a preventive effect on obesity. These *L. salivarius*-mediated changes in functional pathways further support our results that postbiotic supplementation with HK LS 189 can coordinate the gut microbiota and anti-obesity effects.

## Data Availability

The data sets generated and analyzed during the current study are available on the request from the corresponding author.
